# Frontal plane balance during pre-planned and late-cued 90 degree turns while walking

**DOI:** 10.1016/j.jbiomech.2022.111206

**Published:** 2022-06-20

**Authors:** Mitchell Tillman, Janine Molino, Antonia M. Zaferiou

**Affiliations:** a Department of Biomedical Engineering, Stevens Institute of Technology, Hoboken, NJ, USA; b Department of Orthopaedics, Brown University, Providence, RI, USA; Lifespan Biostatistics, Epidemiology, and Research Design Core, Rhode Island Hospital, Providence, RI, USA

**Keywords:** Angular momentum, Turn, Balance, Rotation, Frontal plane, Postural control, Locomotion

## Abstract

This study evaluated frontal-plane dynamic balance control during 90° left turns while walking. Ten healthy young adults performed straight-line gait, pre-planned turns, and turns cued visually (late-cued turns). We quantified rotational balance control via the range of frontal-plane angular momentum (Hf) about the center of mass (COM), and the relative positioning of the COM and the feet using the horizontal distance from the COM to the lateral edge of the base of support (lateral distance) and the mediolateral margin of stability (MOS_ml_). We hypothesized that the Hf range would increase and the lateral distance and MOS_ml_ minima would decrease during each turn type vs. straight-line gait and during late-cued vs. pre-planned turns. We found that the range of Hf was significantly greater during each turn type vs. straight-line gait and during late-cued vs. pre-planned turns. Also, the lateral distance minima were significantly smaller during turns vs. straight-line gait, and during pre-planned vs. late-cued turns. Our hypotheses about MOS_ml_ were partially supported because the MOS_ml_ minima patterns were specific to right or left steps and were not significantly different between straight-line gait and pre-planned turns overall, but the right step’s MOS_ml_ minima were more negative during late-cued vs. pre-planned turns and between either turn and straight-line gait. Finally, we observed slower gait speeds, fewer footfalls, shorter turn phase duration, and different turn strategies used during late-cued vs. pre-planned turns. Overall, these findings reveal multifaceted control of frontal-plane balance during turns encountered during everyday mobility.

## 1. Introduction

Turning while walking comprises up to 50% of all steps taken (Glaister, Bernatz, et al., 2007). Of turning tasks, 90° turning is performed every day as we navigate our built environments. The mechanical objectives of turning include (1) redirection of the body’s velocity vector in the transverse plane, (2) rotation of the body’s facing direction in the transverse plane, and (3) balance maintenance (in the sagittal and frontal planes) throughout the maneuver. The increased dynamic requirements of turns relative to straight-line gait likely challenges balance during the turn (Nolasco et al., 2019), especially in the frontal plane which requires more active balance control than in the sagittal plane (Bauby and Kuo, 2000; Donelan et al., 2004; Mcgeer, 1990; O’Connor and Kuo, 2009; Pandy et al., 2010).

Turns can be executed in a pre-planned or late-cued manner, depending on the environment and movement intent. Pre-planned turns are performed when the cue to turn is perceived early with respect to movement execution. Late-cued turns are required when the cue to turn is perceived later, therefore the movement planning duration is shorter. There is emerging evidence that turn kinematics may be affected by late cues (Dixon et al., 2018; Forsell et al., 2017; Patla et al., 1999).

Balance maintenance requires control of rotation about the body’s center of mass (COM). One common metric for assessing the rotational component of balance is the angular momentum (H) about the COM, also referred to as the whole-body angular momentum. Herr and Popovic showed that H is tightly controlled near zero during straight-line gait in all three planes to facilitate balance maintenance and reduce energy expenditure (Herr and Popovic, 2008). By contrast, turns exhibit asymmetric and relatively large changes in H in all three planes to accomplish the mechanical objectives (Nolasco et al., 2019). Due to the cyclical changes in H about zero during the gait cycle, the range of H – driven by maxima and minima extrema values – is employed to quantify balance control (Herr and Popovic, 2008; Neptune et al., 2019; Silverman et al., 2014; Vistamehr and Neptune, 2021). We expect late cues to further challenge balance during turns or to necessitate strategies that prioritize quickly generating requisite transverse plane momenta, leading to larger ranges of H (although to our knowledge, no studies have quantified H in late-cued turns). For example, the trunk has been observed to rotate more in the frontal plane during late-cued vs. pre-planned turns (Patla et al., 1999). Further, the attentional demands during late-cued turns may be similar to findings that during dual-task walking, people exhibited a “posture-second” strategy (Decker et al., 2016).

Another component of balance control is foot placement relative to the COM horizontal position (Bruijn and Van Dieën, 2018), frequently quantified using the Margin of Stability (MOS) (Hof et al., 2005). The MOS quantifies the distance between the “extrapolated” COM (XCOM) and the nearest edge of the base of support (BOS). In healthy populations, mediolateral MOS (MOS_ml_) during 45°, 90°, and 180° pre-planned turns, and late-cued 180° turns, has been shown to reach more extreme values than during straight-line gait (Dixon et al., 2016; He et al., 2018). Two prior studies of turning while walking have analyzed “lateral distance” between the base of support and horizontal COM position as a measure of balance state. During turns, both the lateral-MOS and the lateral distance decreased relative to straight-line gait (Dixon et al., 2016; Mellone et al., 2016). In comparison to pre-planned turns, late-cued turns have demonstrated an increased maximum center of mass acceleration (Dixon et al., 2018), which may affect the lateral distance. Another study found that “the body midpoint” (as a COM proxy) shifted more towards the stance foot in the direction of the turn (e.g., the left foot during a left turn) during turns than it did during straight-line gait (Courtine and Schieppati 2003). Additionally, Patla et al., found that late-cued turns were initiated with frontal plane trunk rotation towards the direction of the turn, whereas pre-planned turns were initiated by placing the foot towards the direction of the turn (Patla et al., 1999). Therefore, we expect that if this COM translation occurs without a corresponding change in foot placement, it could lead to smaller MOS and lateral distances. Building from this prior work, we expect that the lateral distance and MOS_ml_ will decrease during turns relative to straight line gait and decrease the most during late-cued turns.

The purpose of this study was to understand how frontal-plane balance is regulated during straight-line gait and 90-degree pre-planned and late-cued turns, with respect to rotational control and the relative positioning of the center of mass and feet. We hypothesized that there will be (1) larger ranges of Hf during late-cued and pre-planned turns vs. straight-line gait, (2) decreased minimum lateral distance and MOS_ml_ during late-cued and pre-planned turns vs. straight-line gait, (3) larger ranges of Hf during late-cued vs. pre-planned, and (4) decreased minimum lateral distance and MOS_ml_ during late-cued vs. pre-planned turns.

## 2. Methods

### 2.1. Participants, experimental setup, and procedures

Ten healthy adults (3 females, 7 males; 25.2 ± 4.2 years; 73.9 ± 14.8 kg; 1.79 ± 0.1 m) provided their informed consent to participate in this study as approved by the Institutional Review Board of Stevens Institute of Technology. All participants indicated that they were free of pathologies and pain that would impair their ability to walk and turn during daily life.

**Fig. 1.**
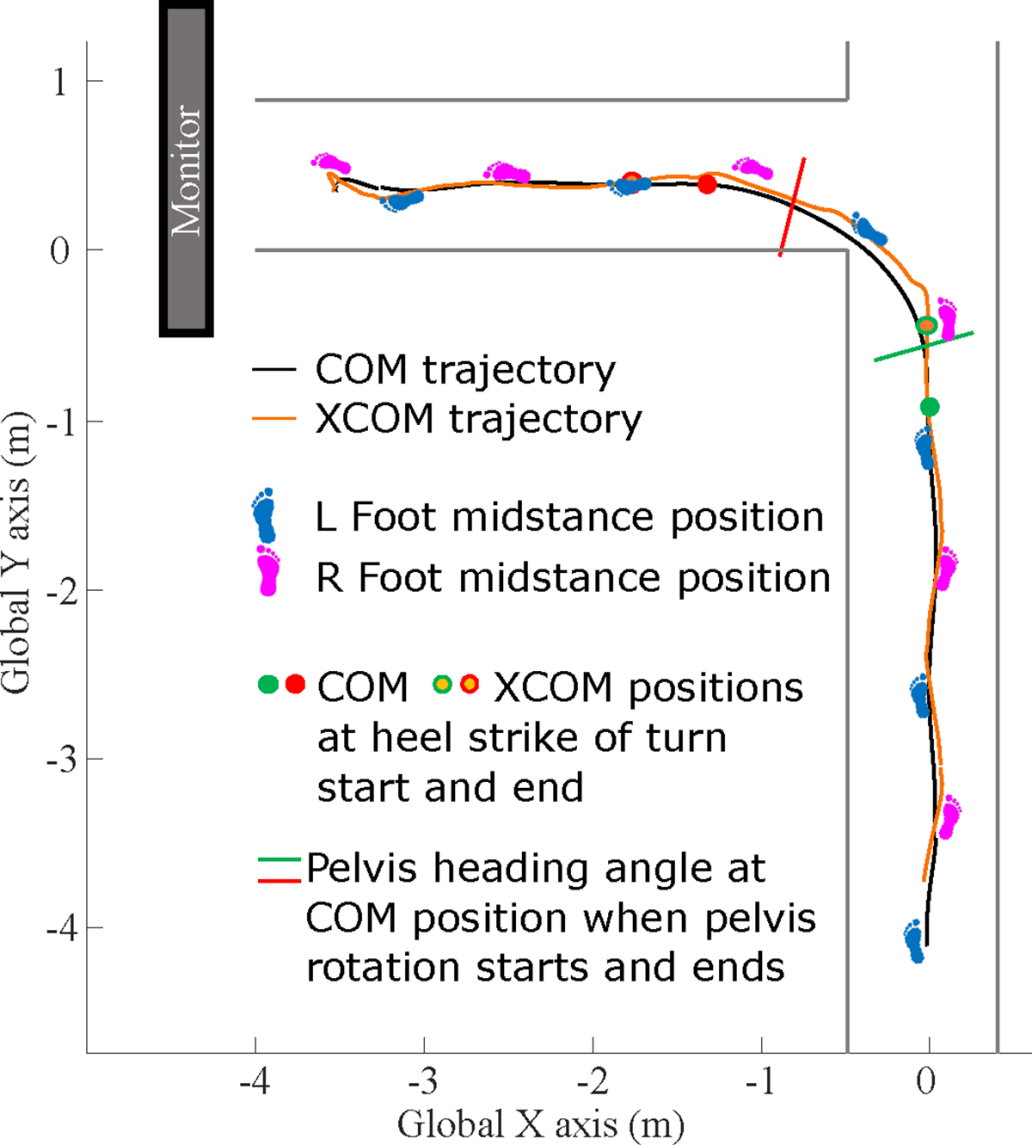
Experimental setup and example center of mass (COM) and extrapolated COM (XCOM) trajectory, footfalls, and turn phase start and end events within the intersection walkway during a pre-planned turn. During late-cued turns, the monitor displayed the cue to turn or continue straight-line gait.

We simulated the conditions of a grocery store using tape on the floor in a T-shape to emulate two aisles 0.91 m wide (Department of Justice 2010) forming a perpendicular intersection (Fig. 1). A 2.03 m diagonal screen at the end of the intersecting aisle served as the aisle’s signage. 61 retroreflective markers were placed on participants to record motion data with optical motion capture (200 fps, Motive 2.2, NaturalPoint, Corvallis, OR, USA) at the following locations: sternum jugular notch; sternum xiphoid process; C7, T2, and T7 vertebrae; as well as left and right: anterior and posterior head; glenohumeral joint; clavicle-acromion joint; humerus lateral epicondyle; posterior aspect of the upper arm; radial and ulnar styloid processes; second and fourth metacarpal; anterior and posterior superior iliac spines; femoral greater trochanter; anterior aspect of the thigh; femoral lateral epicondyle; fibular attachment to the tibia; tibial tuberosity; anterior aspect of the shank; lateral malleolus; first and fifth metatarsal; first distal phalanx; calcaneus.

Participants were asked to imagine that they were in a grocery store walking at a comfortable pace in three contexts: walking straight, pre-planned turns, and late-cued turns. They were instructed to walk as if they were walking down a grocery store aisle with no one in front of them, with people behind them such that they should not stop, and that they were not in a rush. First, they performed five trials of straight-line walking down the 10 m aisle. Next, they performed 10 pre-planned turns, followed by 10 late-cued turns, with 15 s rest periods between trials and five-minute instructional periods prior to each of the three conditions. For both turn conditions, we randomly prescribed with which foot to initiate walking, such that each turn condition included five trials starting with each foot. During the pre-planned turn condition, participants were instructed ahead of time that they should turn 90° left to walk down the intersecting aisle because it contained the item of interest, as though they were familiar with its location in this grocery store. The monitor displayed a large image of the item of interest, green broccoli (Fig. 1).

In the late-cued turn condition, participants knew there was a 50% chance they needed to turn left into the aisle, as though they were unfamiliar with this store and needed to look into the aisle in order to determine to turn. The monitor always started with a black screen, and the participants knew that upon reaching the intersection, the monitor would display either: the green broccoli to cue them to turn, or a “NO” symbol (red circle with a line through it) to cue them to continuing to walk straight. 20 trials were completed, half of which included a late-cued turn.

### 2.2. Kinematic analyses

All marker data were smoothed with a cubic spline filter (MATLAB ‘csaps’ function with the smoothing value set to 0.0005) and no data were gap-filled. Four trials deriving from two subjects were excluded because of missing data due to marker occlusion. To generate the 15-segment whole-body model of each participant (Dumas et al., 2007), first a static trial and a dynamic functional hip joint center calibration trial were performed. Hip joint center positions were obtained following (Schwartz and Rozumalski, 2005) and the shoulders from (Rab et al., 2002). In addition to the balance-specific metrics compared, we computed step and stride length, and stride width according to (Huxham et al., 2006). We also computed the descriptive measures of step and stride duration, turn duration, number of footfalls within a turn, gait speed, and turn strategy. We report the position of the COM relative to the intersection at pelvis rotation onset and cue presentation, the time delay of pelvis rotation onset after cue presentation, and turn strategy (Golyski and Hendershot, 2017).

#### 2.2.1. Phases of interest

In straight-line gait trials, the phase of interest was when the COM was within the center 3 m of the walkway, bounded by heel strike events to ensure complete gait cycles, to avoid transient behavior at gait initiation and termination. In turn trials, all analyses were performed during the turn phase, defined as follows. We used a person-specific threshold based on when the pelvis heading angle exceeded the mean pelvis heading angle plus or minus three times the standard deviation of the pelvis heading angles during straight-line gait trials. The heel strike before the pelvis threshold was reached was the start of the turn phase. The end of the turn was defined by the first heel strike that occurred after the pelvis heading angle reduced below the threshold relative to the new perpendicular direction of travel (-X direction, Fig. 1).

#### 2.2.2. Frontal plane angular momentum (Hf)

Whole-body angular momentum (H) was computed and normalized using methods previously described (Vistamehr and Neptune, 2021). H was projected onto the body-fixed coordinate axes to attain frontal-plane H (Hf). The transverse plane was defined by the global vertical axis, while the frontal plane was defined by the anteriorly-directed pelvis heading in the transverse plane (Dixon et al., 2014; Fino et al., 2015; Glaister, Orendurff, et al., 2007). For each trial, we computed the maximum and minimum Hf as well as the range (Hf maximum minus Hf minimum).

#### 2.2.3. Base of support (BOS)

We quantified the BOS by which markers are below a height threshold as defined during quiet standing per participant. Four markers on each foot (heel, metatarsophalangeal joint 1, metatarsophalangeal joint 5, and distal phalange 2) were used to compute the BOS. To minimize the occurrence of only one marker at heel-strike contributing to a non-anatomical triangular BOS, we buffered the forefoot and hindfoot BOS areas with ellipse planes of best fit. Because the COM velocity vector was always anteriorly directed, we simplified by only using the front foot’s lateral edge during the double support phases. The BOS is the horizontal-plane convex hull outline of the front foot’s markers that are below their threshold height obtained during quiet standing, plus a 1 cm height tolerance. Heel strike and toe-off gait events were detected using the relative positioning of the foot markers and pelvis (Zeni et al., 2008) modified for turning gait (Ulrich et al., 2019). We included the small vertical buffer of 1 cm to avoid situations where the BOS was incorrect due only to change in shape of the footwear during heel-strike and toe-off subphases.

#### 2.2.4. Lateral distance

**Fig. 2.**
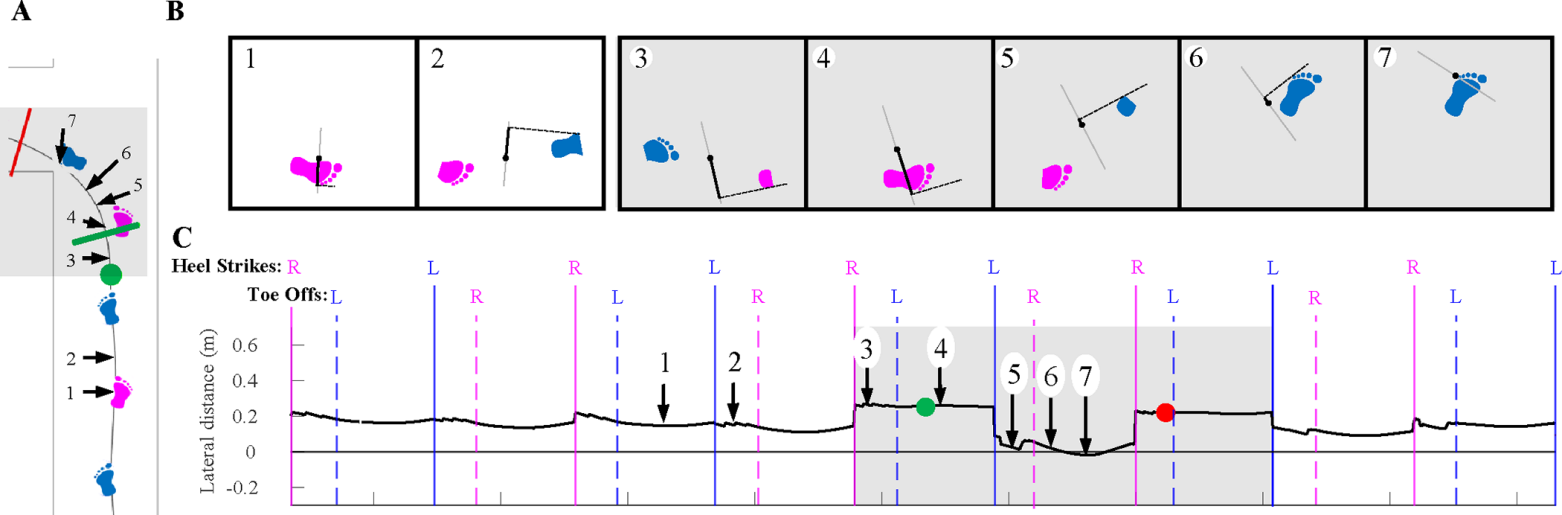
An example of the lateral distance metric during a pre-planned turn (same trial as Fig. 1). (**A**) center of mass (COM) position within the walkway at select gait events, (**B**) cartoons of lateral distance computation at select gait events, (**C**) timeseries of the lateral distance, select gait events are labelled. The gray shaded areas indicate the turn phase and the vertical lines indicate either heel strike (solid) or toe-off (dashed) events for the right (pink) or left (blue) foot. (For interpretation of the references to colour in this figure legend, the reader is referred to the web version of this article.)

Lateral distance was defined as the horizontal distance between the COM and the BOS lateral edge, where lateral was defined by the pelvis mediolateral axis (Fig. 2). To compare across participants, the lateral distance was normalized to leg length (trochanter height).

Because only the lateral edges of the BOS were considered, when the COM is within the BOS or medial to it – right of the left foot or left of the right foot –the lateral distance metric is positive. When the COM is lateral of the lateral edge of the BOS, this lateral distance metric is negative (event 7, Fig. 2). In each trial, the maximum and minimum across both feet, as well as during left and right steps individually, during the turn were compared.

#### 2.2.5. Mediolateral Margin of Stability (MOS_ml_)

The MOS_ml_ was computed from the same BOS position, COM position, and gait event data used in the lateral distance computation. However, both the medial and lateral edges of the BOS were considered when computing the distance from the XCOM to the edges of the BOS. The extrapolated COM (XCOM) position was obtained using an effective pendulum length of 1.34 times the trochanteric height (Massen and Kodde, 1979). During the turn, maxima and minima across both feet, as well as for during left and right steps individually, were extracted for analysis.

### 2.3. Statistical analyses

Differences in frontal plane angular momentum, lateral distance, MOS_ml_, and spatiotemporal descriptors across straight-line gait and turn conditions were examined using linear mixed models that included random intercepts for study participant, random slopes for trial number nested within condition and study participant, and fixed effects for study condition (*lmer* function in R version 4.1.2) (R Core Team, 2021). Mixed models were chosen because they allowed for the appropriate handling of repeated measurements within study participants. Pairwise comparisons between study tasks were estimated within the regression models via orthogonal contrasts. Residuals were examined for all models to ensure that all assumptions were met. The Holm test was used to correct for multiple comparisons and maintain a two-tailed familywise alpha at 0.05 across the hypotheses we tested. An adjusted p-value < 0.05 was used to determine statistical significance (Westfall et al., 2014).

## 3. Results

### 3.1. Spatiotemporal measures

Spatiotemporal results and p-values are included in Table 1 and Supplemental Document 1. Minimum, median, and maximum gait speed significantly decreased from straight-line gait to pre-planned turns and to late-cued turns.

### 3.2. Frontal plane angular momentum (Hf)

**Fig. 3.**
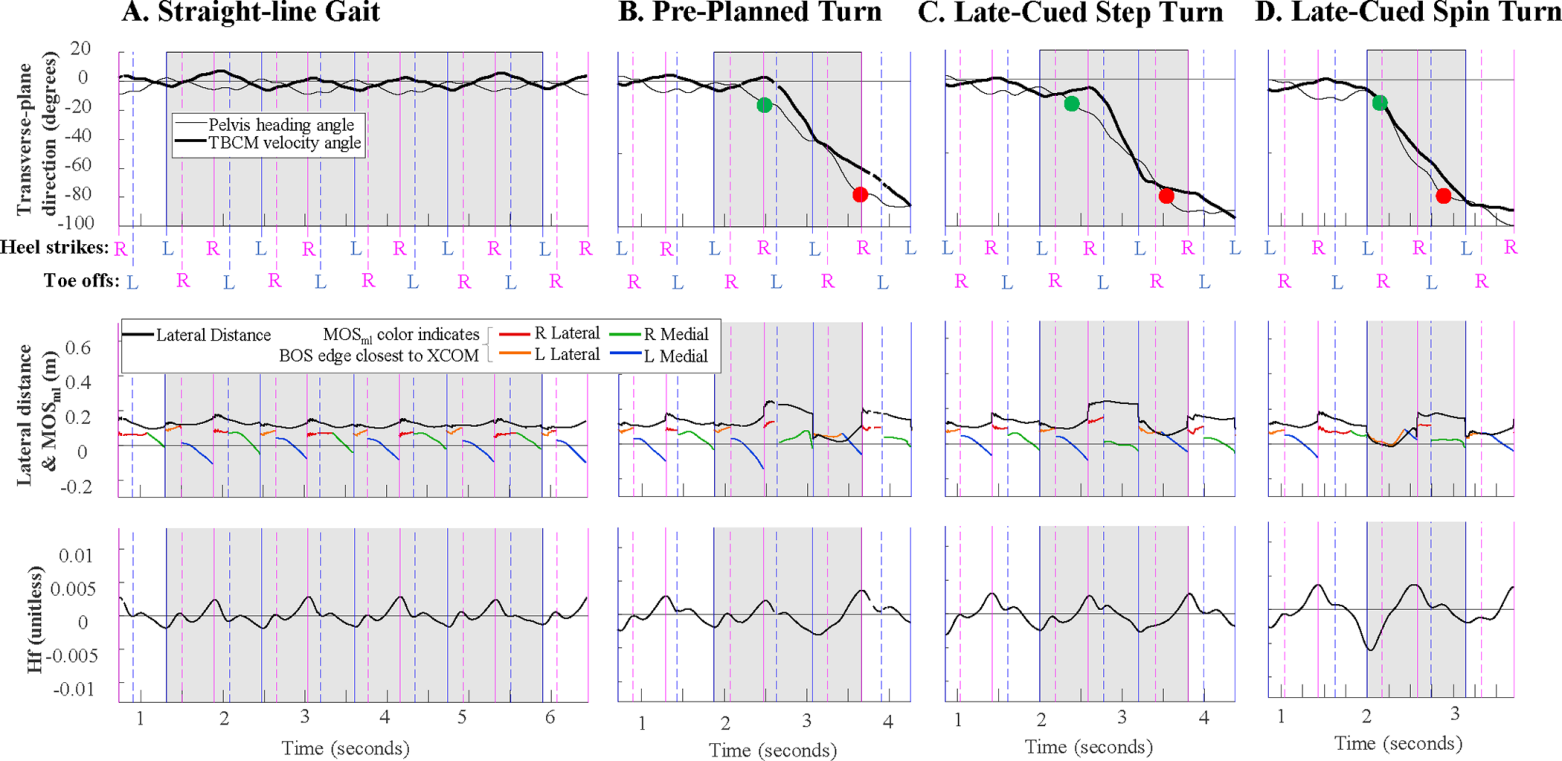
Example timeseries during (**A**) straight-line gait, (**B**) pre-planned turn, (**C**) late-cued step turn, and (**D**) late-cued spin turn. Top to bottom: (**top**) Transverse-plane center of mass velocity direction (thick line) and pelvis heading angle (thin line) displayed such that 0° is aligned with global +Y and −90° is aligned with −X (leftward is negative), (**middle**) Lateral distance and MOS, where each color of the MOS reflects the closest edge of the BOS, (**bottom**) Frontal-plane angular momentum (Hf). The gray shaded areas indicate the phase of interest and the vertical lines indicate either heel strike (solid) or toe-off (dashed) events for the right (pink) or left (blue) foot. The green and red circles mark the start and end of pelvis rotation, respectively. These trials are from participant 5, as indicated in Figs. 4–6. (For interpretation of the references to colour in this figure legend, the reader is referred to the web version of this article.)

**Fig. 4.**
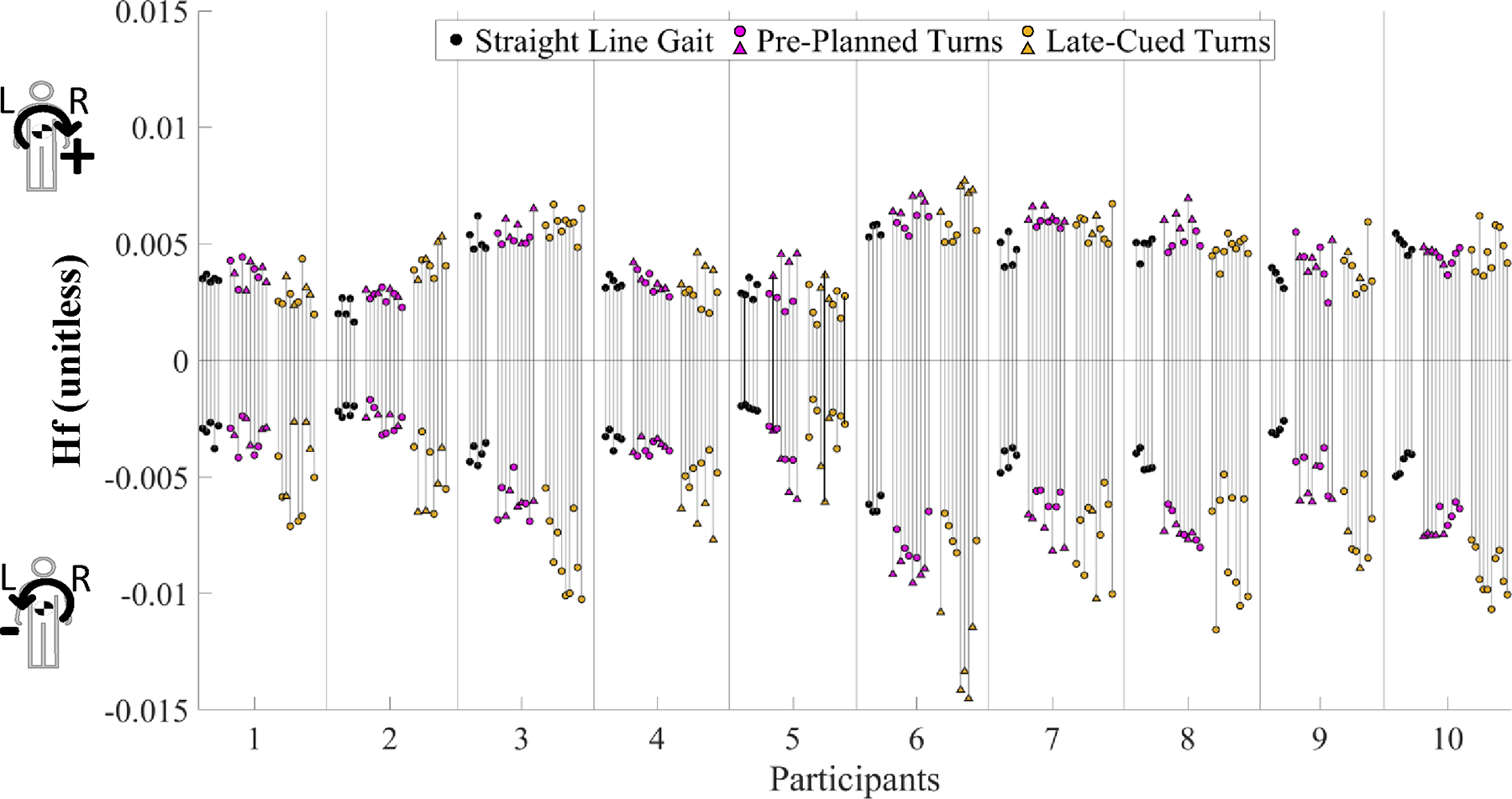
Frontal-plane angular momentum (Hf) minima and maxima during the phase of interest for each trial for all conditions and participants. Triangle marks indicate spin turns and circular marks indicate step turns. Example trials included in Fig. 3 are emphasized here with black bars overlaid on their ranges instead of grey.

Hf range was significantly smaller during straight-line gait vs. pre-planned turns (p < 0.0001) and late-cued turns (p < 0.0001) and during pre-planned vs. late-cued turns (p = 0.005) (Table 2, Fig. 3, Fig. 4). These changes in Hf range were associated with significantly smaller minima between all task types (p < 0.0001), while maxima were only significantly greater during pre-planned turns vs. straight-line gait (p = 0.008).

### 3.3. Lateral distance

**Fig. 5.**
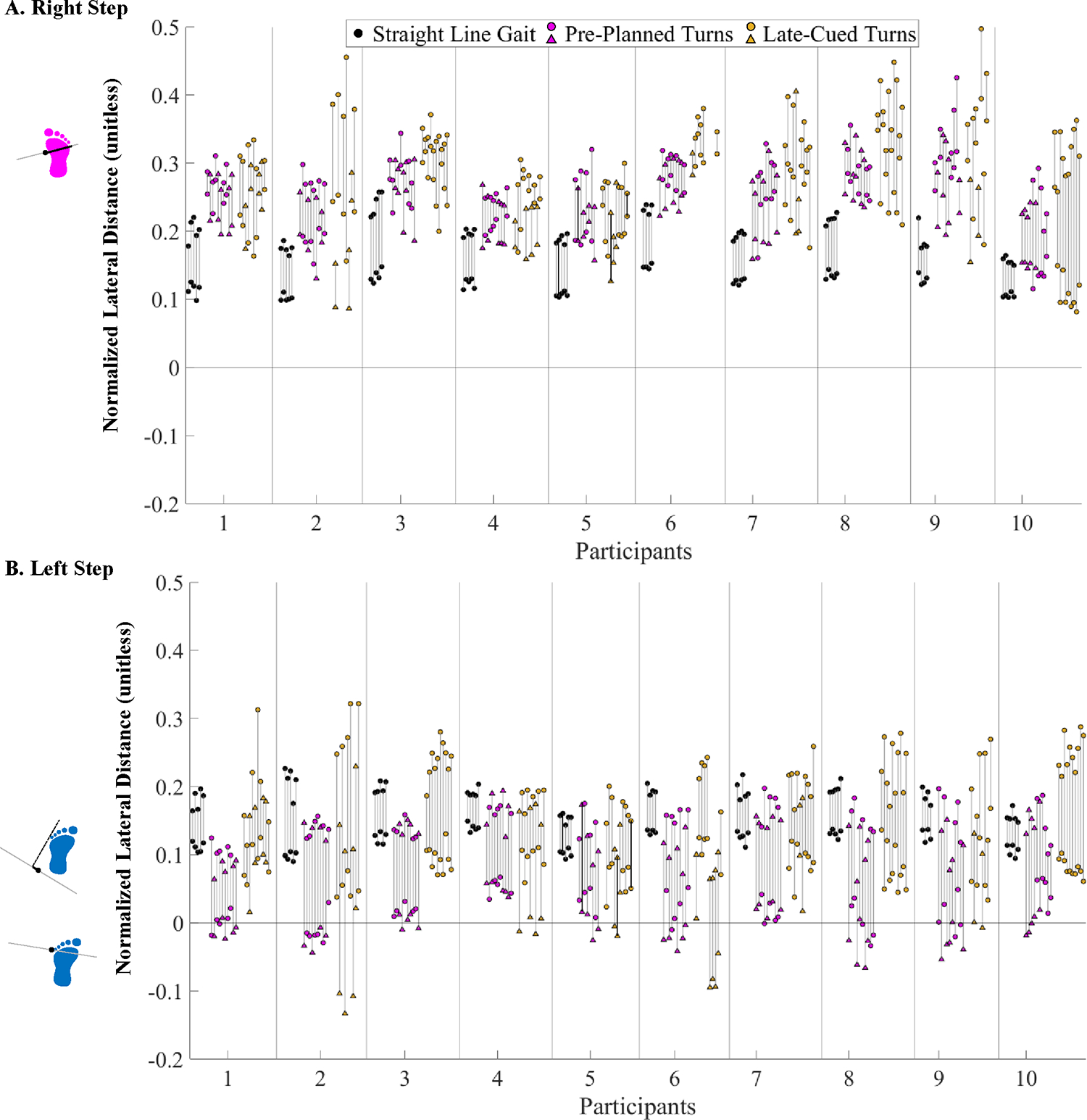
Lateral distance minima and maxima during (**A**) Right Steps and (**B**) Left Steps (normalized to participant leg length) during the phase of interest for each trial for all conditions and participants. Triangle marks indicate spin turns and circular marks indicate step turns. Example trials included in Fig. 3 are emphasized here with black bars between maxima and minima instead of grey.

The lateral distance minima were significantly larger during straight-line gait vs. pre-planned (p < 0.0001) and late-cued turns (p < 0.0001) and during late-cued vs. pre-planned turns (p < 0.0001) (Table 2, Fig. 3, Fig. 5). During turns, the lateral distance maxima always occurred during a right step, if a right step was included in the turn phase (all turn trials except five late-cued turn trials included a right step). Right step lateral distance minima were smaller during straight-line gait vs. pre-planned (p < 0.0001) and late-cued turns (p < 0.0001) (Fig. 5A, Table 2). During turns, the lateral distance minima occurred during a left step for all trials (Fig. 7). The left step lateral distance minima were larger during straight-line gait vs. pre-planned (p < 0.0001) and late-cued turns (p < 0.0001) and during late-cued vs. pre-planned turns (p < 0.0001) (Fig. 5B, Table 2).

### 3.4. Mediolateral margin of stability (MOS_ml_)

**Fig. 6.**
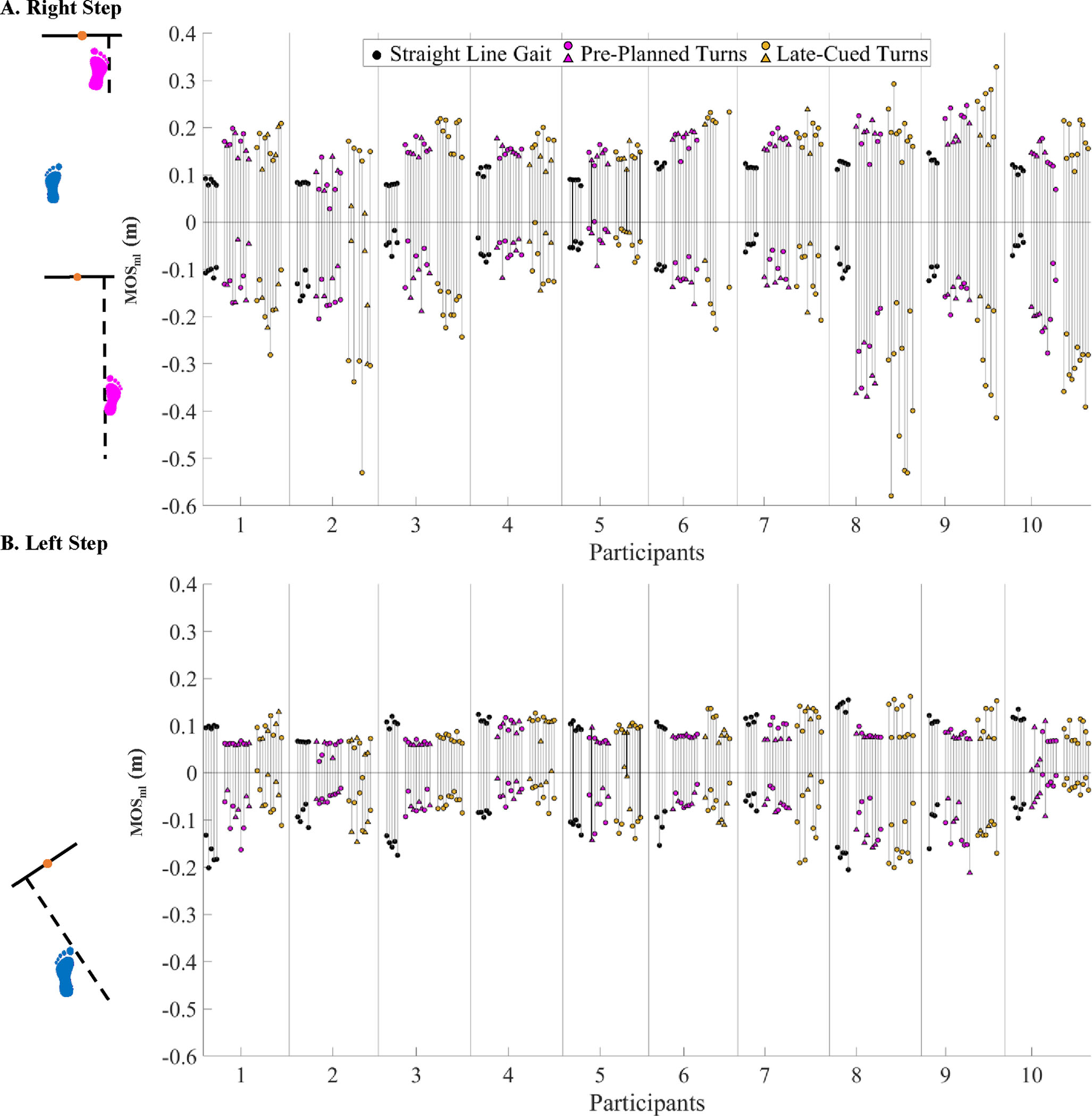
Mediolateral Margin of Stability (MOS_ml_) during (**A**) Right Steps and (**B**) Left Steps (normalized to participant leg length) during the phase of interest for each trial for all conditions and participants. Triangle marks indicate spin turns and circular marks indicate step turns. Example trials included in Fig. 3 are emphasized here with black bars between maxima and minima instead of grey.

**Fig. 7.**
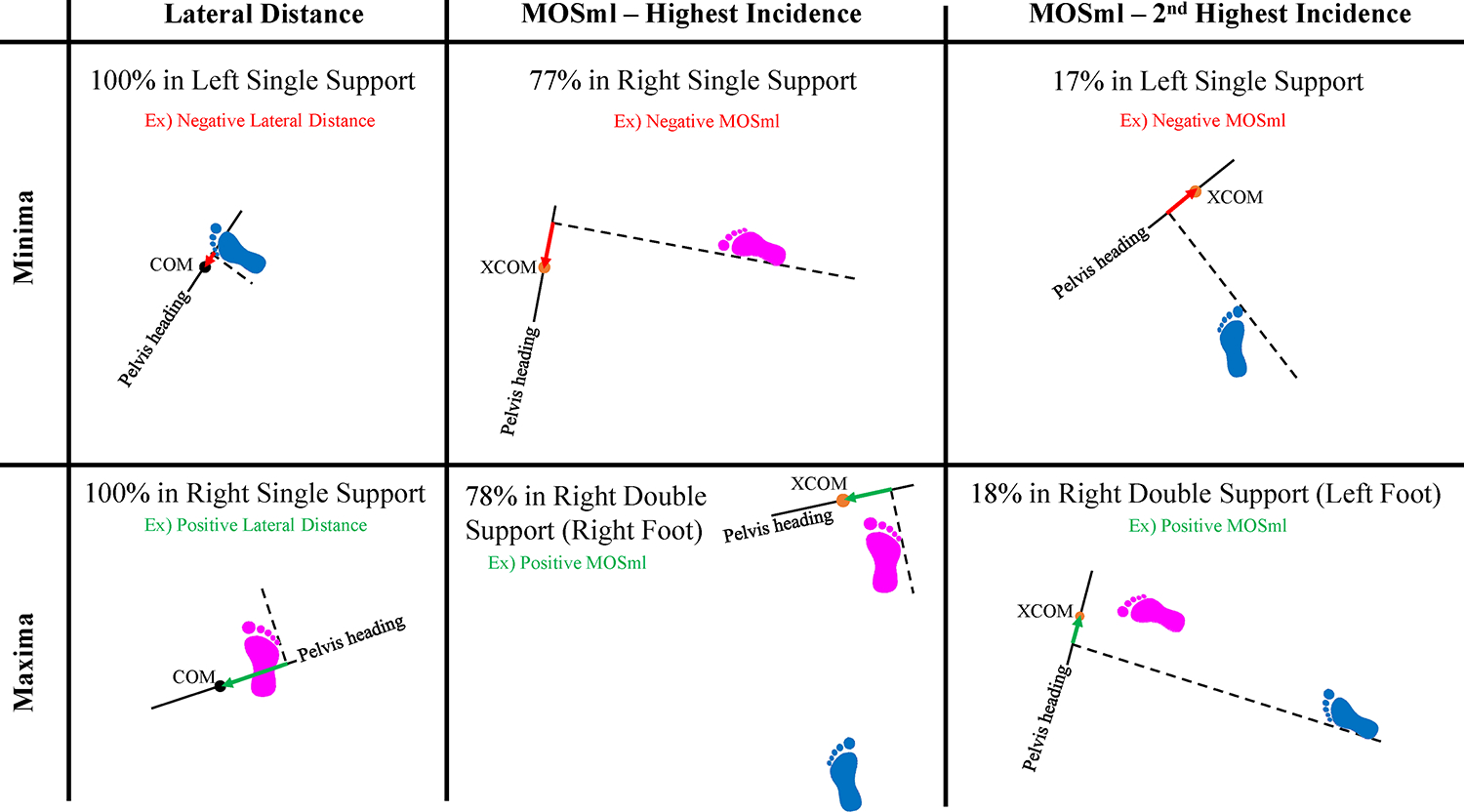
The percent incidence and footfall context for minima and maxima lateral distance and MOS_ml_ that occurred most frequently.

The MOS_ml_ minima were significantly less negative during straight-line gait vs. late-cued turns (p < 0.0001) and during pre-planned vs. late-cued turns (p = 0.002) (Table 2, Fig. 3, Fig. 6). During turns, the footfall context for MOS_ml_ minima and maxima were mixed across gait phases (Fig. 7). The MOS_ml_ minima were generally during right single stance phase (77% incidence) when the XCOM was left of the right foot’s medial BOS edge. About 17% of MOS_ml_ minima were during left single support (in ~14% of trials XCOM was to the right of left foot medial BOS edge, and ~3% of trials XCOM was to the left of the left foot’s lateral BOS edge). Fig. 7 describes the context for MOS_ml_ maxima, except for one trial when the maximum was during left double support.

During right steps, MOS_ml_ minima were less negative during straight-line gait vs. pre-planned (p = 0.0002) and late-cued (p < 0.0001) and during pre-planned vs. late-cued turns (p = 0.003) (Table 2, Fig. 6A). Left step MOS_ml_ minima were more negative during straight-line gait vs. pre-planned (p < 0.0001) and late-cued turns (p = 0.0001) (Table 2, Fig. 6B).

## 4. Discussion

This study focused on understanding the frontal plane dynamic balance control during straight-line gait, leftward pre-planned turns, and leftward late-cued turns, quantified by Hf, lateral distance, and MOS_ml_. Supporting our hypotheses about Hf, its range was greater during each turn type vs. straight-line gait and during late-cued vs. pre-planned turns. Our hypotheses about lateral distance were partially supported because the lateral distance minima were smaller during turns vs. straight-line gait, but they were also smaller during pre-planned vs. late-cued turns. Our hypotheses about MOS_ml_ were partially supported because the MOS_ml_ minima were not significantly different between straight-line gait and pre-planned turns overall, but were specific to right or left step. The left step’s MOS_ml_ minima were smaller negative values during pre-planned turns vs. straight-line gait, but the right step’s MOS_ml_ minima were more negative during late-cued vs. pre-planned turns and between either turn and straight-line gait. Finally, relative to pre-planned turns, we observed changes in spatiotemporal patterns during late-cued turns including slower gait speeds, fewer footfalls, shorter turn phase duration, and differing turn strategies. During late-cued turns, 73% were step turns, whereas, during pre-planned turns, step or spin turns each occurred 50% of the time and were linked to the foot with which participants started walking in all trials except two (Supplemental Table 1).

Greater Hf ranges used during turns vs. straight-line gait is supported by related research that found that turns increased average Hf (Nolasco et al., 2019) and that angular momentum can become “highly unregulated” (Farrell and Herr, 2008). Our findings of greater Hf range during late-cued turns are only adjacently supported by prior research (no prior research directly compared Hf in late-cued vs. pre-planned turns). With fewer footfalls and shorter turn durations observed during late-cued turns in this study, the body needs to change its heading direction more during each footfall. Larger changes in heading direction have been linked to increased frontal-plane trunk rotation (Courtine and Schieppati, 2003), which may align with larger Hf range observed during late-cued turns. Other research has found that slower straight-line gait speeds have exhibited increased Hf extrema, and we observed slower gait speeds during late-cued vs. pre-planned turns (Bennett et al., 2010; Silverman and Neptune, 2011). Although, in our preliminary exploration accounting for gait speed as a covariate still demonstrated smaller Hf minima during late-cued vs. pre-planned turns (Supplemental Document 1).

During turns, lateral distance minima, which occurred during left steps, were smaller than they were during straight-line gait. MOS_ml_ minima generally occurred during the right step and were more negative during turns vs. straight-line gait. During turns, both minima decreased relative to straight line gait as the COM or XCOM were situated leftward within the body’s base of support, as found previously (Conradsson et al., 2018; He et al., 2018; Taylor et al., 2005; Xu et al., 2004) and consistent with the mechanical objectives of generating leftward COM momentum.

**Fig. 8.**
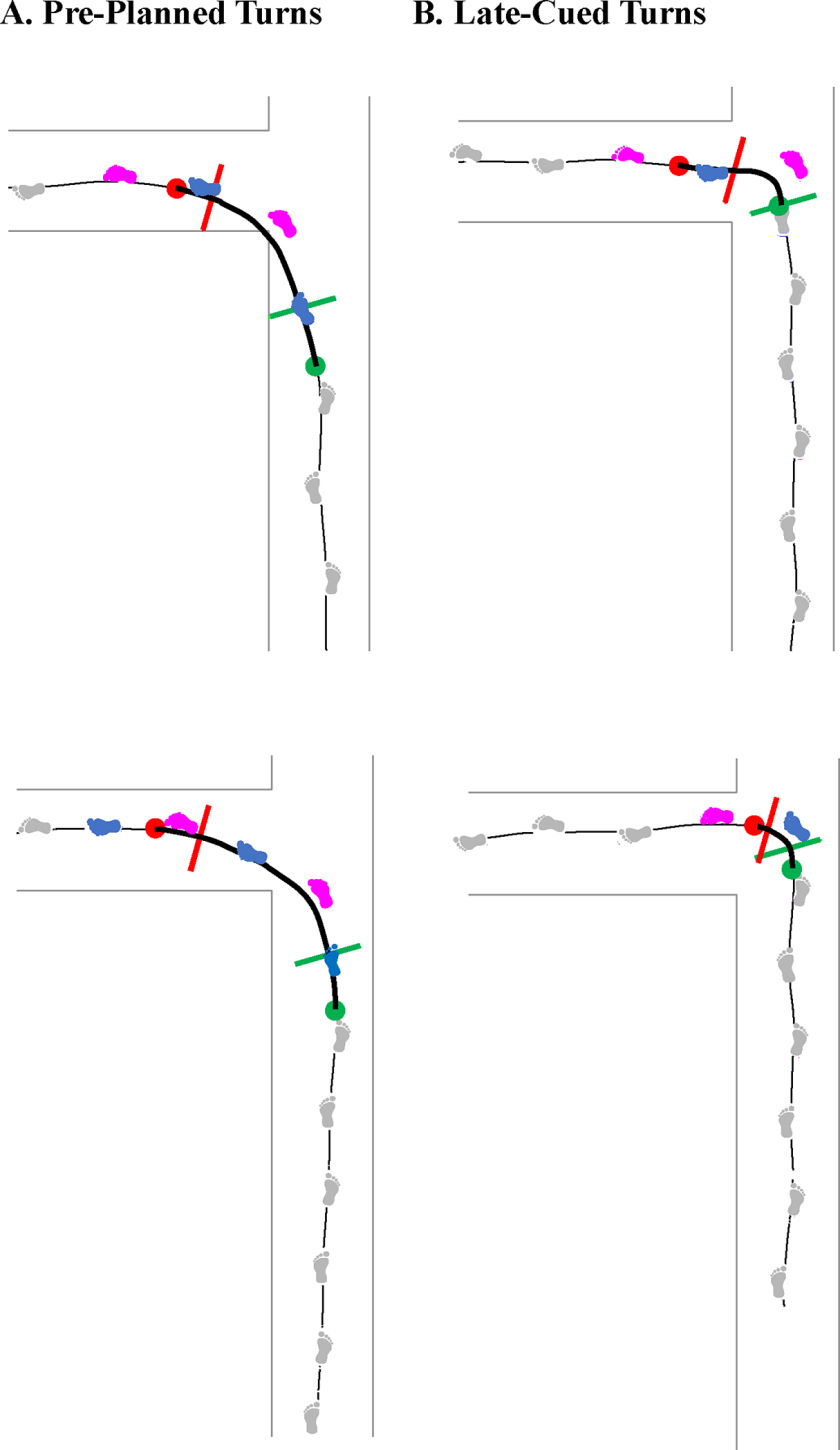
Example trials displaying the wide variety of stepping patterns and numbers of footfalls used during the turn phase (foot cartoons in color) to accomplish (**A**) pre-planned and (**B**) late-cued turns.

Spatiotemporal results as well as emerging observations of differing turn strategies provide context for why the lateral distance minima were greater and MOS_ml_ minima were smaller during late-cued vs. pre-planned turns. We observed faster gait speeds, longer turn durations, and more footfalls used to accomplish pre-planned turns vs. late-cued turns. Additionally, as exemplified in Fig. 8, we observed sharper turns during late-cued vs. pre-planned turns (though this is not explicitly compared in this study). During pre-planned turns, the gait speed and turn radius were similar to that used during a circular gait task previously studied, which demonstrated the COM situated towards the inside of the turn’s interior foot, which would be associated with a small and negative lateral distance (Orendurff et al., 2006). Similar to previous findings about gait speed, late-cued turns in this study used lower gait speeds and larger lateral distance minima than turns performed at higher speeds (Orendurff et al., 2006; Xu et al., 2004). Previously, the percent times that the XCOM and COM were outside of the lateral edge of the BOS increased during turns at faster gait speed (Mellone et al., 2016) which relates to our observation of larger lateral distance minima and smaller MOS_ml_ minima during faster pre-planned turns. In contrast to these prior studies, late-cued turns of the present study used smaller turn radii, as the nature of our late-cued experimental condition enforced planning and executing the turn with less time and less space within the intersection. As in the pre-planned condition, larger turn radii can support faster gait speeds (Brown et al., 2021; Yamaguchi et al., 2017). Finally, prior findings that peak COM acceleration at the apex of the turn increased during late-cued turns (Dixon et al., 2018), may relate to a larger velocity component contributing to the smaller left foot MOS_ml_ minima we observed during late-cued turns.

Upon further exploration, we found that 50% of pre-planned and 73% of late-cued turns were “step turns”, which indicates that the right foot is used as the primary “redirection” footfall (Hase and Stein, 1999; Taylor et al., 2005). These turn strategy findings are aligned with some previous findings for preplanned (Conradsson et al., 2017:20) and unplanned turns (Patla et al., 1999), but not all previous research supported the predominance of step turns during late-cued turns (Conradsson et al., 2017; Dixon et al., 2019). The incidence of turn strategies reported across studies of late-cued turns can be specific to experimental cue conditions and turn strategy computation methods. During late-cued turns, if the right foot was the primary redirection footfall, the lateral distance minima occurring during left step turns are not as small as when the left foot is the primary redirection footfall in a spin turn. Similar to our finding of larger lateral distance minima, step turns are considered more stable (Hase and Stein, 1999; Taylor et al., 2005). Additionally, during trials when spin turns were used during the late-cued turn condition (triangle markers in Figs. 4–6) the lateral distance minima were smaller than those observed during pre-planned turns. By contrast, Hf extrema appear independent of turn strategy (though not empirically tested).

This study has several limitations to note. Despite best efforts to replicate the ecological characteristics of a grocery store in the lab settings, the constraints imposed by motion capture cameras required minimal visual obstruction, so participants were able to look at the screen throughout the approach (unlike a grocery store aisle), though they were instructed not to. Second, the space constraints of the lab required a short (4 m) walkway exiting the turn that may have imposed a velocity reduction requirement during the turning tasks, and we only examined left turns to reduce the duration of the experiment. Further, though we randomized within condition block, we did not randomize the condition blocks, so there could be an order effect between conditions. Also, our turn phase definition relied on pelvis rotation, when other preparatory adjustments may have preceded pelvis rotation. We acknowledge that our results are specific to the turn phase and turn strategy computational choices, as well as our experimental design, including the variable nature of the late-cue timing.

This study opens many future research questions. For example, we would like to understand how the control of frontal plane balance relates to control in other planes and how balance during turns is controlled by different balance-impaired populations. A more thorough analysis of the effects of gait speed on frontal plane balance measurements is prompted by our initial explorations of gait speed as a covariate. Similarly, a thorough analysis of how the gait phase context when the cue was provided and how that affected turning strategies is warranted, as in (Hase and Stein, 1999).

## Supplementary Material

Supplemental Document 1

## Figures and Tables

**Table 1:** Group-level median (IQR) values for spatiotemporal parameters of interest. Global Y position is relative to the coordinate system included in Figure 1, such that the intersection begins at Y=0m. Group level p-values from post hoc pairwise comparisons conducted via orthogonal contrasts within mixed models are included and are bolded when significant.

Parameter		Median (IQR)			Post Hoc Pairwise Comparisons conducted via orthogonal contrasts within mixed models		

		Straight	Pre-Planned	Late-Cued	Straight vs. Pre-Planned	Straight vs. Late-Cued	Pre-Planned vs. Late-Cued

**Step Length (m)**	Median	0.75 (0.65, 0.83)	0.70 (0.61, 0.74)	0.58 (0.50, 0.63)	**<0.0001**	**<0.0001**	**<0.0001**

**Stride Length (m)**	Median	1.49 (1.30, 1.65)	1.37 (1.19, 1.47)	1.23 (0.99, 1.35)	**0.0001**	**<0.0001**	**<0.0001**

**Stride Width (m)**	Minimum	0.09 (0.07, 0.11)	0.10 (0.05, 0.15)	0.07 (0.04, 0.12)	0.99	0.99	0.99
	Median	0.11 (0.10, 0.13)	0.19 (0.17, 0.22)	0.15 (0.07, 0.21)	**<0.0001**	**0.003**	**0.002**
	Maximum	0.13 (0.11, 0.14)	0.28 (0.24, 0.33)	0.20 (0.07, 0.34)	**<0.0001**	**0.0007**	**0.0007**

**Step Duration (s)**	Median	0.57 (0.55, 0.59)	0.57 (0.55, 0.61)	0.60 (0.58, 0.63)	**0.03**	**<0.0001**	**0.03**

**Stride Duration (s)**	Median	1.14 (1.10, 1.17)	1.15 (1.11, 1.22)	1.18 (1.11, 1.29)	0.16	**0.004**	0.16

**Gait Speed (m/s)**	Minimum	1.17 (1.03, 1.29)	1.00 (0.83, 1.12)	0.57 (0.47, 0.64)	**<0.0001**	**<0.0001**	**<0.0001**
	Median	1.29 (1.12, 1.40)	1.15 (1.00, 1.27)	0.93 (0.82, 1.03)	**<0.0001**	**<0.0001**	**<0.0001**
	Maximum	1.52 (1.32, 1.64)	1.37 (1.20, 1.53)	1.26 (1.12, 1.35)	**<0.0001**	**<0.0001**	**<0.0001**

**Number of footfalls**	Median	8.00 (7.00, 8.25)	4.00 (4.00, 4.00)	3.00 (3.00, 4.00)	**<0.0001**	**<0.0001**	**<0.0001**

**Phase Duration (s)**	Median	3.89 (3.67, 4.25)	1.71 (1.63, 1.82)	1.40 (1.18, 1.70)	**<0.0001**	**<0.0001**	**<0.0001**

**COM global Y position relative to intersection at pelvis rotation onset (m)**	Median	-	−0.23 (0.13, 0.37)	0.35 (−0.50, −0.21)	-	-	**<0.0001**

**COM global Y position relative to intersection at cue (m)**	Median	-	-	−0.14 (0.09)	-	-	-

**Pelvis rotation onset after cue time (sec)**	Median	-	-	0.52 (0.14)	-	-	-

**Cue delivered at % of gait cycle (right to right foot)**	Range			4 – 97	-	-	-

**Table 2:** Group-level estimated marginal means for the study’s primary outcome variables. Group level p-values from post hoc pairwise comparisons conducted via orthogonal contrasts within mixed models are included and are bolded when significant.

Parameter		Estimated Marginal Mean (95% CI)			Post Hoc Pairwise Comparisons conducted via orthogonal contrasts within mixed models		

		Straight	Pre-Planned	Late-Cued	Straight vs. Pre-Planned	Straight vs. Late-Cued	Pre-Planned vs. Late-Cued

**Hf (x10^−3^)**	Minimum	−3.69 (−4.90, −2.49)	−5.31 (−6.52, −4.11)	−6.71 (−7.91, −5.50)	**<0.0001**	**<0.0001**	**<0.0001**
	Maximum	4.11 (3.33, 4.90)	4.60 (3.81, 5.39)	4.34 (3.56, 5.13)	**0.008**	0.21	0.21
	Range	7.81 (5.87, 9.75)	9.90 (7.96, 11.84)	11.02 (9.08, 12.96)	**<0.0001**	**<0.0001**	**0.005**

**Lateral Distance – Left Step**	Minimum	0.12 (0.10, 0.14)	0.01 (−0.01, 0.02)	0.06 (0.05, 0.08)	**<0.0001**	**<0.0001**	**<0.0001**
	Maximum	0.19 (0.17, 0.20)	0.14 (0.13, 0.16)	0.20 (0.18, 0.21)	**<0.0001**	0.31	**<0.0001**

**Lateral Distance – Right Step**	Minimum	0.12 (0.10, 0.15)	0.22 (0.19, 0.24)	0.23 (0.20, 0.26)	**<0.0001**	**<0.0001**	0.058
	Maximum	0.20 (0.18, 0.22)	0.28 (0.26, 0.30)	0.32 (0.30, 0.34)	**<0.0001**	**<0.0001**	**<0.0001**

**MOS – Left Step (m)**	Minimum	−0.11 (−0.13, −0.09)	−0.07 (−0.09, −0.05)	−0.08 (−0.10, −0.06)	**<0.0001**	**0.0001**	0.26
	Maximum	0.11 (0.09, 0.12)	0.07 (0.06, 0.08)	0.09 (0.08, 0.11)	**<0.0001**	**0.002**	**<0.0001**

**MOS – Right Step (m)**	Minimum	−0.07 (−0.13, −0.03)	−0.14 (−0.19, −0.09)	−0.19 (−0.24, −0.14)	**0.0002**	**<0.0001**	**0.003**
	Maximum	0.11 (0.09, 0.13)	0.16 (0.14, 0.18)	0.18 (0.16, 0.20)	**<0.0001**	**<0.0001**	**0.001**

**Lateral Distance**	Minimum	0.12 (0.10, 0.13)	0.01 (−0.01, 0.02)	0.06 (0.05, 0.08)	**<0.0001**	**<0.0001**	**<0.0001**
	Maximum	0.21 (0.18, 0.23)	0.28 (0.26, 0.30)	0.31 (0.29, 0.34)	**<0.0001**	**<0.0001**	**0.001**

**MOS (m)**	Minimum	−0.12 (−0.17, −0.07)	−0.15 (−0.19, −0.10)	−0.20 (−0.24, −0.15)	0.055	**<0.0001**	**0.002**
	Maximum	0.12 (0.09, 0.13)	0.16 (0.14, 0.18)	0.17 (0.15, 0.19)	**<0.0001**	**<0.0001**	**0.02**
