# Supplementary material for: Frontal plane balance during pre-planned and late-cued 90 degree turns while walking
Source: J Biomech. Author manuscript; Available in PMC 2023 Dec 15. (PMC10722562; doi:10.1016/j.jbiomech.2022.111206)
Supplement: Supplemental Document 1 [file NIHMS1948386-supplement-Supplemental_Document_1.docx]

**Supplemental Document 1**

**This document includes the following supplemental information:**

1. **Turn strategy (Supplemental Table 1)**
2. **Summary of spatiotemporal results and discussion.**
3. **Lateral Distance and MOSml results figures when they are not separated by right or left steps.**
4. **Covariate Exploration**

**I. Supplemental Table 1:** Turn strategies used (step or spin) and their overall percent (%) incidence per participant depending on the foot they used to initiate the trial during each turn condition of pre-planned and late-cued turns. Overall percent (%) incidence of turn strategy are also provided across all trials for each pre-planned or late-cued turn conditions.

| **Participant** | **Pre-planned turns** | | | **Late-cued turns** | | |
| --- | --- | --- | --- | --- | --- | --- |
|  | **Turn strategy when Right foot started the trial** | **Turn strategy when Left foot started the trial** | **% Step turns** | **Turn strategy when Right foot started the trial** | **Turn strategy when Left foot started the trial** | **% Step Turns** |
| **1** | Spin | Step | 50 | Step | Spin* | 60 |
| **2** | Step* | Spin* | 50 | Spin | Step | 55 |
| **3** | Step | Spin | 50 | Step | Step | 100 |
| **4** | Step | Spin | 50 | Step | Spin* | 60 |
| **5** | Spin | Step | 50 | Mixed | Mixed | 70 |
| **6** | Step | Spin | 50 | Spin | Step | 50 |
| **7** | Spin | Step | 50 | Mixed | Mixed | 80 |
| **8** | Step | Spin | 50 | Step | Step | 100 |
| **9** | Spin | Step | 50 | Mixed | Mixed | 75 |
| **10** | Step | Spin | 50 | Step | Step | 100 |
| **Overall** |  |  | **50%** |  |  | **73%** |

* Indicates that one trial was an exception to this pattern.

**II.a. Spatiotemporal Results**

All spatiotemporal outcomes and p-values are shown in **Table 1** in the main text. Minimum, median, and maximum gait speed changed significantly between all comparisons, walking slower in late-cued vs. pre-planned and either turn type vs. straight-line gait. Median stride and step lengths decreased significantly from straight-line gait to pre-planned to late-cued turns. Median and maximum stride width decreased significantly from pre-planned to late-cued turns to straight-line gait (minimum stride width was not significantly different between any condition). Step duration increased from straight-line to pre-planned to late-cued turns, while stride duration was only significantly greater during late-cued vs. straight-line gait. The number of footfalls during the turn decreased significantly in late-cued vs. pre-planned turns. During pre-planned turns the pelvis began rotating before the intersection was reached, and in late-cued turns rotation began after reaching the intersection. When the late cue was provided, the median COM position was -0.14 m (global Y-direction, **Figure 1**) relative to the intersection and the median time the pelvis began rotating was 0.52 s after the late-cue was provided.

**II.b. Spatiotemporal Discussion**

Spatiotemporal trends provide context for other balance-related results: late-cued turn phase durations were shorter, gait speed was slower, and the number of footfalls was generally fewer. Due to the nature of the visual cue being provided when the body entered the intersection at different phases of the gait cycle, it is sensible that we observed greater variability in turn balance measurements as the body needed to adjust to the cue while in different configurations to complete the turn quickly. Participants could have leveraged a lower gait speed as a strategy to allow them to successfully complete the late-cued turn by allowing enough time at a lower gait speed to process and plan action from receiving the visual cue without passing the intersection. Additionally, lower gait speeds could facilitate using a smaller turn radius (tradeoff between speed and turn radius). An exploration of gait speed metrics as covariates is included in section III of this supplemental document.

**
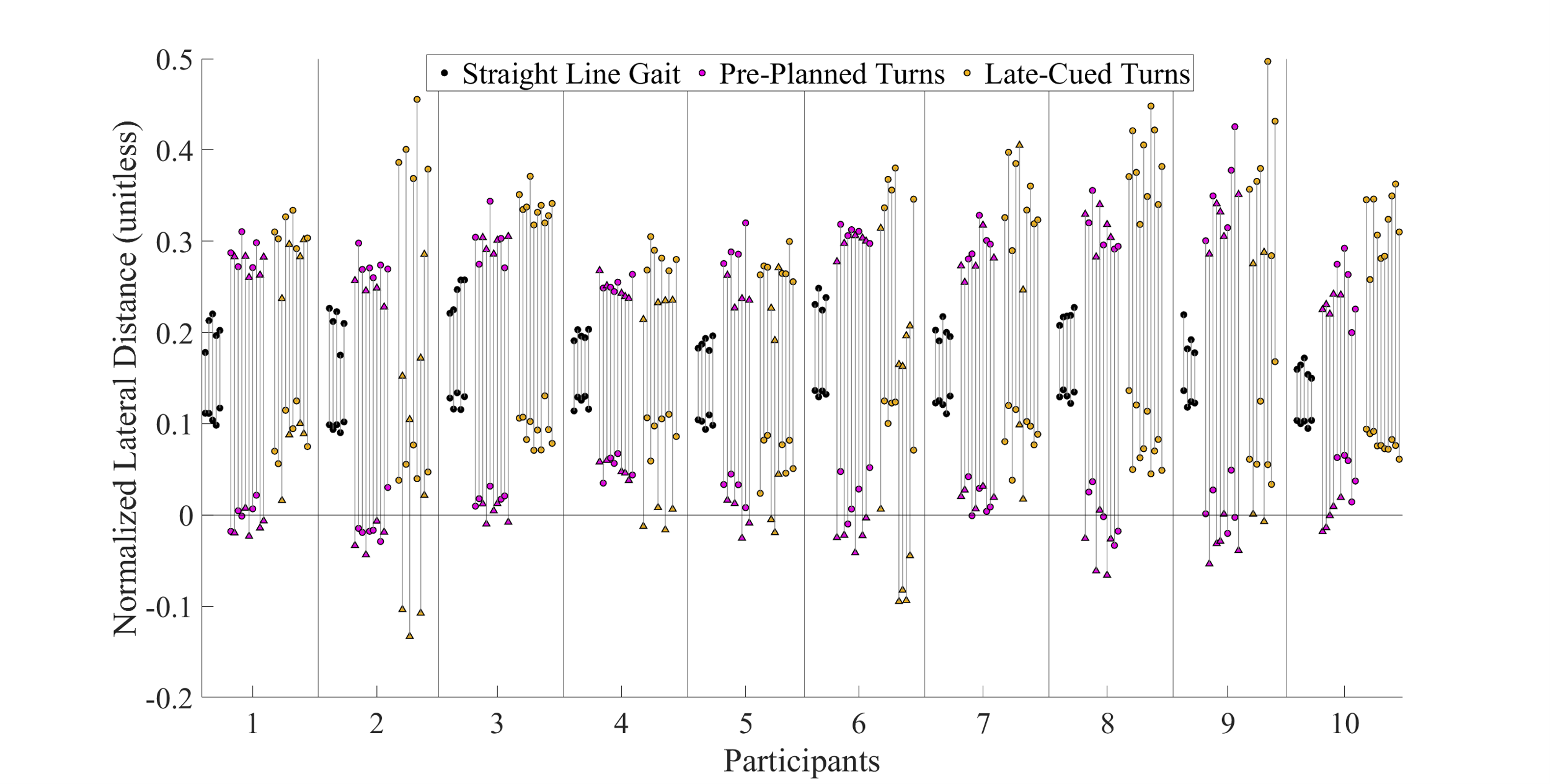
III.a. Lateral Distance (not separated by right or left steps)**

**III.b. MOSml (not separated by right or left steps)**

**
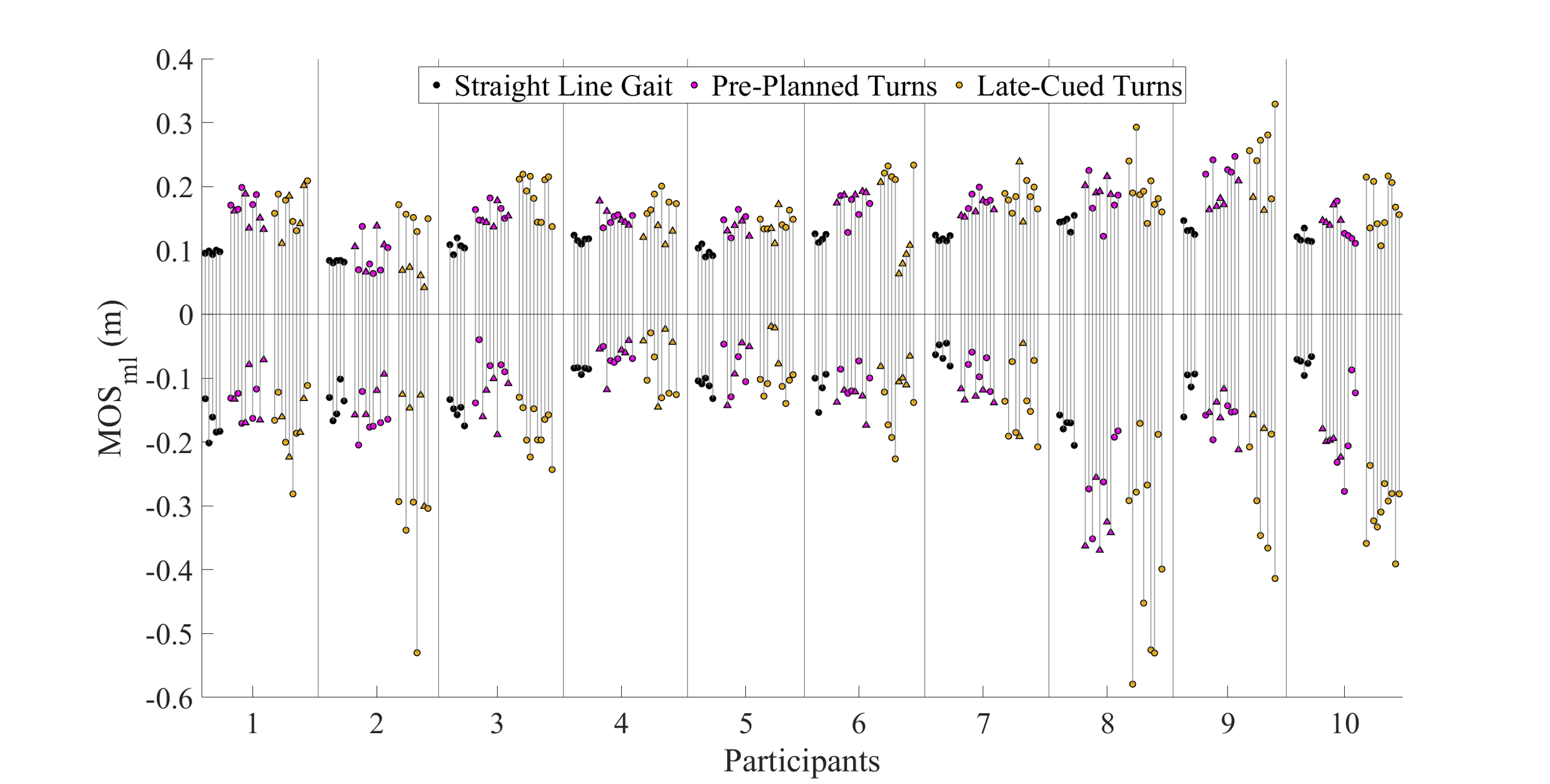
**

**IV. Covariate Exploration**

Exploratory analyses added gait speed (minimum, median, maximum), trial number, and starting foot (for turn conditions only) as fixed effects to the above models in order to assess their effect on the study findings.

**Supplemental Table 2:** Group-level estimated marginal means for the study’s primary outcome variables analyzed using mixed models with *minimum gait* speed as a covariate. Group level p-values from post hoc pairwise comparisons conducted via orthogonal contrasts within mixed models are included and are bolded when significant.

| Parameter | | Estimated Marginal Mean (95% CI) | | | Post Hoc Pairwise Comparisons Conducted Via Orthogonal Contrasts within Mixed Models | | |
| --- | --- | --- | --- | --- | --- | --- | --- |
|  |  | **Straight** | **Pre-Planned** | **Late-Cued** | **Straight vs. Pre-Planned** | **Straight vs. Late-Cued** | **Pre-Planned vs. Late-Cued** |
| Hf (x10^-3^) | **Minimum** | -2.62 (-3.94, -1.30) | -4.87 (-6.09, -3.64) | -7.55 (-8.84, -6.27) | **<0.0001** | **<0.0001** | **<0.0001** |
|  | **Maximum** | 3.80 (2.97, 4.64) | 4.48 (3.69, 5.28) | 4.60 (3.78, 5.42) | **0.001** | **0.048** | 0.89 |
|  | **Range** | 6.36 (4.30, 8.42) | 9.32 (7.36, 11.28) | 12.16 (10.14, 14.19) | **<0.0001** | **<0.0001** | **<0.0001** |
| Lateral Distance – Left Foot | **Minimum** | 0.17 (0.15, 0.19) | 0.03 (0.01, 0.04) | 0.02 (0.00, 0.04) | **<0.0001** | **<0.0001** | 0.94 |
|  | **Maximum** | 0.23 (0.21, 0.25) | 0.16 (0.14, 0.18) | 0.16 (0.14, 0.18) | **<0.0001** | **0.0001** | 0.99 |
| Lateral Distance – Right Foot | **Minimum** | 0.13 (0.10, 0.16) | 0.22 (0.19, 0.25) | 0.23 (0.20, 0.26) | **<0.0001** | **<0.0001** | 0.80 |
|  | **Maximum** | 0.23 (0.20, 0.26) | 0.29 (0.27, 0.32) | 0.30 (0.27, 0.33) | **<0.0001** | **0.0002** | 0.97 |
| MOSml (m) – Left Foot | **Minimum** | -0.10 (-0.12, -0.07) | -0.07 (-0.09, -0.05) | -0.10 (-0.12, -0.07) | **0.002** | 0.99 | **0.049** |
|  | **Maximum** | 0.12 (0.10, 0.13) | 0.08 (0.06, 0.09) | 0.08 (0.07, 0.10) | **<0.0001** | **0.0005** | 0.45 |
| MOSml (m) – Right Foot | **Minimum** | -0.10 (-0.16, -0.04) | -0.15 (-0.20, -0.10) | -0.17 (-0.22, -0.11) | **0.03** | 0.15 | 0.81 |
|  | **Maximum** | 0.11 (0.09, 0.14) | 0.16 (0.14, 0.18) | 0.17 (0.15, 0.19) | **<0.0001** | **0.0001** | 0.63 |
| Lateral Distance | **Minimum** | 0.16 (0.14, 0.18) | 0.02 (0.01, 0.04) | 0.02 (0.00, 0.04) | **<0.0001** | **<0.0001** | 0.96 |
|  | **Maximum** | 0.23 (0.20, 0.26) | 0.29 (0.27, 0.32) | 0.29 (0.27, 0.32) | **<0.0001** | **0.004** | 0.99 |
| MOS (m) | **Minimum** | -0.15 (-0.21, -0.09) | -0.16 (-0.21, -0.11) | -0.17 (-0.23, -0.12) | 0.82 | 0.76 | 0.88 |
|  | **Maximum** | 0.12 (0.10, 0.15) | 0.16 (0.14, 0.18) | 0.17 (0.14, 0.19) | **<0.0001** | **0.006** | 0.92 |

**Supplemental Table 3:** Group-level estimated marginal means for the study’s primary outcome variables analyzed using mixed models with *median gait* speed as a covariate. Group level p-values from post hoc pairwise comparisons conducted via orthogonal contrasts within mixed models are included and are bolded when significant.

| Parameter | | Estimated Marginal Mean (95% CI) | | | Post Hoc Pairwise Comparisons Conducted Via Orthogonal Contrasts within Mixed Models | | |
| --- | --- | --- | --- | --- | --- | --- | --- |
|  |  | **Straight** | **Pre-Planned** | **Late-Cued** | **Straight vs. Pre-Planned** | **Straight vs. Late-Cued** | **Pre-Planned vs. Late-Cued** |
| Hf (x10^-3^) | **Minimum** | -3.81 (-5.07, -2.54) | -5.34 (-6.56, -4.11) | -6.63 (-7.87, -5.38) | **<0.0001** | **<0.0001** | **0.0007** |
|  | **Maximum** | 4.13 (3.32, 4.94) | 4.60 (3.81, 5.40) | 4.33 (3.53, 5.13) | **0.03** | 0.67 | 0.29 |
|  | **Range** | 7.97 (5.96, 9.97) | 9.93 (7.97, 11.90) | 10.91 (8.93, 12.90) | **<0.0001** | **<0.0001** | 0.07 |
| Lateral Distance – Left Foot | **Minimum** | 0.11 (0.09, 0.13) | 0.00 (-0.01, 0.02) | 0.07 (0.05, 0.09) | **<0.0001** | **0.004** | **<0.0001** |
|  | **Maximum** | 0.18 (0.16, 0.19) | 0.14 (0.13, 0.16) | 0.20 (0.19, 0.22) | **0.0004** | 0.08 | **<0.0001** |
| Lateral Distance – Right Foot | **Minimum** | 0.11 (0.08, 0.13) | 0.21 (0.19, 0.24) | 0.24 (0.22, 0.27) | **<0.0001** | **<0.0001** | **0.004** |
|  | **Maximum** | 0.19 (0.17, 0.21) | 0.28 (0.26, 0.30) | 0.33 (0.31, 0.35) | **<0.0001** | **<0.0001** | **<0.0001** |
| MOSml (m) – Left Foot | **Minimum** | -0.09 (-0.11, -0.07) | -0.07 (-0.08, -0.05) | -0.10 (-0.11, -0.08) | **0.009** | 0.97 | **0.004** |
|  | **Maximum** | 0.11 (0.09, 0.12) | 0.07 (0.06, 0.08) | 0.09 (0.08, 0.11) | **<0.0001** | 0.09 | **<0.0001** |
| MOSml (m) – Right Foot | **Minimum** | -0.05 (-0.10, -0.01) | -0.14 (-0.18, -0.09) | -0.20 (-0.25, -0.16) | **<0.0001** | **<0.0001** | **0.0003** |
|  | **Maximum** | 0.10 (0.08, 0.12) | 0.16 (0.14, 0.18) | 0.18 (0.16, 0.20) | **<0.0001** | **<0.0001** | **0.0008** |
| Lateral Distance | **Minimum** | 0.10 (0.09, 0.12) | 0.00 (-0.01, 0.02) | 0.07 (0.05, 0.09) | **<0.0001** | **0.009** | **<0.0001** |
|  | **Maximum** | 0.18 (0.16, 0.20) | 0.28 (0.26, 0.30) | 0.33 (0.31, 0.35) | **<0.0001** | **<0.0001** | **<0.0001** |
| MOS (m) | **Minimum** | -0.09 (-0.13, -0.05) | -0.14 (-0.18, -0.10) | -0.22 (-0.26, -0.18) | **0.005** | **<0.0001** | **<0.0001** |
|  | Maximum | 0.10 (0.08, 0.12) | 0.16 (0.14, 0.17) | 0.18 (0.16, 0.20) | **<0.0001** | **<0.0001** | **0.001** |

**Supplemental Table 4:** Group-level estimated marginal means for the study’s primary outcome variables analyzed using mixed models with both *minimum and median* gait speed as covariates. Group level p-values from post hoc pairwise comparisons conducted via orthogonal contrasts within mixed models are included and are bolded when significant.

| Parameter | | Estimated Marginal Mean (95% CI) | | | Post Hoc Pairwise Comparisons Conducted Via Orthogonal Contrasts within Mixed Models | | |
| --- | --- | --- | --- | --- | --- | --- | --- |
|  |  | **Straight** | **Pre-Planned** | **Late-Cued** | **Straight vs. Pre-Planned** | **Straight vs. Late-Cued** | **Pre-Planned vs. Late-Cued** |
| Hf (x10^-3^) | **Minimum** | -2.67 (-4.06, -1.29) | -4.74 (-6.03, -3.44) | -7.61 (-8.96, -6.26) | **<0.0001** | **<0.0001** | **<0.0001** |
|  | **Maximum** | 3.83 (2.97, 4.68) | 4.46 (3.64, 5.27) | 4.62 (3.78, 5.45) | **0.003** | **0.049** | 0.80 |
|  | **Range** | 6.47 (4.32, 8.61) | 9.18 (7.12, 11.23) | 12.22 (10.11, 14.33) | **<0.0001** | **<0.0001** | **<0.0001** |
| Lateral Distance – Left Foot | **Minimum** | 0.17 (0.15, 0.19) | 0.04 (0.02, 0.05) | 0.01 (-0.01, 0.03) | **<0.0001** | **<0.0001** | **0.03** |
|  | **Maximum** | 0.23 (0.21, 0.25) | 0.17 (0.15, 0.18) | 0.15 (0.14, 0.17) | **<0.0001** | **<0.0001** | 0.26 |
| Lateral Distance – Right Foot | **Minimum** | 0.13 (0.10, 0.16) | 0.23 (0.20, 0.25) | 0.22 (0.19, 0.25) | **<0.0001** | **<0.0001** | 0.87 |
|  | **Maximum** | 0.23 (0.20, 025) | 0.30 (0.28, 0.32) | 0.29 (0.27, 0.31) | **<0.0001** | **0.0001** | 0.55 |
| MOSml (m) – Left Foot | **Minimum** | -0.10 (-0.12, -0.07) | -0.07 (-0.09, -0.05) | -0.09 (-0.11, -0.07) | **0.008** | 0.93 | 0.18 |
|  | **Maximum** | 0.12 (0.10, 0.13) | 0.08 (0.07, 0.09) | 0.08 (0.07, 0.10) | **<0.0001** | **0.0002** | 0.71 |
| MOSml (m) – Right Foot | **Minimum** | -0.10 (-0.15, -0.05) | -0.16 (-0.20, -0.12) | -0.16 (-0.20, -0.11) | **0.002** | 0.18 | 0.99 |
|  | **Maximum** | 0.12 (0.09, 0.14) | 0.17 (0.15, 0.19) | 0.16 (0.14, 0.19) | **<0.0001** | **0.001** | 0.97 |
| Lateral Distance | **Minimum** | 0.17 (0.15, 0.18) | 0.04 (0.02, 0.05) | 0.01 (-0.01, 0.03) | **<0.0001** | **<0.0001** | **0.04** |
|  | **Maximum** | 0.23 (0.20, 0.25) | 0.30 (0.29, 0.32) | 0.29 (0.27, 0.31) | **<0.0001** | **0.0008** | 0.35 |
| MOS (m) | **Minimum** | -0.14 (-0.19, -0.10) | -0.17 (-0.20, -0.13) | -0.17 (-0.21, -0.13) | 0.30 | 0.64 | 0.99 |
|  | **Maximum** | 0.12 (0.10, 0.15) | 0.17 (0.15, 0.19) | 0.16 (0.14, 0.18) | **<0.0001** | **0.01** | 0.85 |

**Supplemental Table 5:** Group-level estimated marginal means for the study’s primary outcome variables analyzed using mixed models with *trial* as a covariate. Group level p-values from post hoc pairwise comparisons conducted via orthogonal contrasts within mixed models are included and are bolded when significant.

| Parameter | | Estimated Marginal Mean (95% CI) | | | Post Hoc Pairwise Comparisons Conducted Via Orthogonal Contrasts within Mixed Models | | |
| --- | --- | --- | --- | --- | --- | --- | --- |
|  |  | **Straight** | **Pre-Planned** | **Late-Cued** | **Straight vs. Pre-Planned** | **Straight vs. Late-Cued** | **Pre-Planned vs. Late-Cued** |
| Hf (x10^-3^) | **Minimum** | -3.83 (-5.05, -2.62) | -5.42 (-6.63, -4.21) | -6.82 (-8.03, -5.61) | **<0.0001** | **<0.0001** | **<0.0001** |
|  | **Maximum** | 4.12 (3.33, 4.91) | 4.61 (3.81, 5.40) | 4.35 (3.56, 5.14) | **0.008** | 0.32 | 0.24 |
|  | **Range** | 7.96 (6.01, 9.91) | 10.03 (8.08, 11.98) | 11.16 (9.21, 13.11) | **<0.0001** | **<0.0001** | **0.01** |
| Lateral Distance – Left Foot | **Minimum** | 0.12 (0.10, 0.14) | 0.01 (-0.01, 0.02) | 0.06 (0.05, 0.08) | **<0.0001** | **<0.0001** | **<0.0001** |
|  | **Maximum** | 0.19 (0.17, 0.20) | 0.14 (0.13, 0.16) | 0.20 (0.18, 0.21) | **<0.0001** | 0.56 | **<0.0001** |
| Lateral Distance – Right Foot | **Minimum** | 0.12 (0.09, 0.15) | 0.21 (0.19, 0.24) | 0.23 (0.20, 0.26) | **<0.0001** | **<0.0001** | 0.14 |
|  | **Maximum** | 0.20 (0.18, 0.22) | 0.28 (0.26, 0.31) | 0.32 (0.30, 0.34) | **<0.0001** | **<0.0001** | **<0.0001** |
| MOSml (m) – Left Foot | **Minimum** | -0.11 (-0.14, -0.09) | -0.07 (-0.09, -0.05) | -0.08 (-0.10, -0.06) | **<0.0001** | **0.0002** | 0.51 |
|  | **Maximum** | 0.11 (0.09, 0.12) | 0.07 (0.06, 0.08) | 0.09 (0.08, 0.11) | **<0.0001** | **0.006** | **<0.0001** |
| MOSml (m) – Right Foot | **Minimum** | -0.08 (-0.13, -0.03) | -0.14 (-0.19, -0.09) | -0.19 (-0.24, -0.14) | **0.0003** | **<0.0001** | **0.008** |
|  | **Maximum** | 0.10 (0.08, 0.13) | 0.16 (0.14, 0.18) | 0.18 (0.16, 0.20) | **<0.0001** | **<0.0001** | **0.004** |
| Lateral Distance | **Minimum** | 0.12 (0.10, 0.13) | 0.01 (-0.01, 0.02) | 0.06 (0.05, 0.08) | **<0.0001** | **<0.0001** | **<0.0001** |
|  | **Maximum** | 0.21 (0.18, 0.23) | 0.28 (0.26, 0.30) | 0.32 (0.29, 0.34) | **<0.0001** | **<0.0001** | **0.004** |
| MOS (m) | **Minimum** | -0.12 (-0.17, -0.07) | -0.15 (-0.19, -0.10) | -0.20 (-0.24, -0.15) | 0.14 | **<0.0001** | **0.004** |
|  | **Maximum** | 0.11 (0.09, 0.13) | 0.16 (0.14, 0.18) | 0.17 (0.15, 0.19) | **<0.0001** | **<0.0001** | **0.046** |

**Supplemental Table 6:** Group-level estimated marginal means for the study’s primary outcome variables analyzed using mixed models with *starting foot* as a covariate. Group level p-values from post hoc pairwise comparisons conducted via orthogonal contrasts within mixed models are included and are bolded when significant.

| Parameter | | Estimated Marginal Mean (95% CI) | | | Post Hoc Pairwise Comparisons Conducted Via Orthogonal Contrasts within Mixed Models | | |
| --- | --- | --- | --- | --- | --- | --- | --- |
|  |  | **Straight** | **Pre-Planned** | **Late-Cued** | **Straight vs. Pre-Planned** | **Straight vs. Late-Cued** | **Pre-Planned vs. Late-Cued** |
| Hf (x10^-3^) | **Minimum** | - | -5.38 (-6.80, -3.96) | -6.91 (-8.33, -5.50) | - | - | **<0.0001** |
|  | **Maximum** | - | 4.54 (3.69, 5.40) | 4.42 (3.57, 5.28) | - | - | 0.25 |
|  | **Range** | - | 9.92 (7.70, 12.10) | 11.33 (9.11, 13.60) | - | - | **<0.0001** |
| Lateral Distance – Left Foot | **Minimum** | - | 0.01 (-0.01, 0.02) | 0.06 (0.04, 0.08) | - | - | **<0.0001** |
|  | **Maximum** | - | 0.14 (0.12, 0.15) | 0.20 (0.18, 0.22) | - | - | **<0.0001** |
| Lateral Distance – Right Foot | **Minimum** | - | 0.21 (0.18, 0.25) | 0.23 (0.20, 0.26) | - | - | **0.03** |
|  | **Maximum** | - | 0.28 (0.26, 0.31) | 0.32 (0.30, 0.35) | - | - | **<0.0001** |
| MOSml (m) – Left Foot | **Minimum** | - | -0.07 (-0.10, -0.05) | -0.08 (-0.10, -0.06) | - | - | 0.14 |
|  | **Maximum** | - | 0.07 (0.06, 0.08) | 0.09 (0.08, 0.10) | - | - | **<0.0001** |
| MOSml (m) – Right Foot | **Minimum** | - | -0.13 (-0.19, -0.07) | -0.19 (-0.26, -0.13) | - | - | **<0.0001** |
|  | **Maximum** | - | 0.16 (0.14, 0.18) | 0.18 (0.15, 0.20) | - | - | **0.0001** |
| Lateral Distance | **Minimum** | - | 0.01 (-0.01, 0.02) | 0.06 (0.04, 0.08) | - | - | **<0.0001** |
|  | **Maximum** | - | 0.28 (0.26, 0.31) | 0.31 (0.29, 0.34) | - | - | **<0.0001** |
| MOS (m) | **Minimum** | - | -0.14 (-0.20, -0.09) | -0.20 (-0.26, -0.15) | - | - | **<0.0001** |
|  | **Maximum** | - | 0.16 (0.14, 0.18) | 0.17 (0.15, 0.19) | - | - | **0.008** |
